# Bulked Segregant RNA-Seq Reveals Distinct Expression Profiling in Chinese Wheat Cultivar Jimai 23 Responding to Powdery Mildew

**DOI:** 10.3389/fgene.2020.00474

**Published:** 2020-05-27

**Authors:** Tong Zhu, Liru Wu, Huagang He, Jiancheng Song, Mengshu Jia, Liancheng Liu, Xiaolu Wang, Ran Han, Liping Niu, Wenxiao Du, Xu Zhang, Wenrui Wang, Xiao Liang, Haosheng Li, Jianjun Liu, Hongxing Xu, Cheng Liu, Pengtao Ma

**Affiliations:** ^1^School of Life Sciences, Yantai University, Yantai, China; ^2^School of Food and Biological Engineering, Jiangsu University, Zhenjiang, China; ^3^Beijing Biomics Technology Company Limited, Beijing, China; ^4^Crop Research Institute, Shandong Academy of Agricultural Sciences, Jinan, China; ^5^State Key Laboratory of Hybrid Rice, College of Life Sciences, Wuhan University, Wuhan, China; ^6^State Key Laboratory of Crop Stress Adaptation and Improvement, School of Life Sciences, Henan University, Kaifeng, China

**Keywords:** wheat, powdery mildew, BSR-Seq, expression profiling, DEG

## Abstract

Wheat powdery mildew, caused by *Blumeria graminis* f. sp. *tritici* (*Bgt*), is one of the most destructive fungal diseases threatening global wheat production. Host resistance is well known to be the most efficient method to control this disease. However, the molecular mechanism of wheat powdery mildew resistance (*Pm*) is still unclear. To analyze the molecular mechanism of *Pm*, we used the resistant wheat cultivar Jimai 23 to investigate its potential resistance components and profiled its expression in response to powdery mildew infection using bulked segregant RNA-Seq (BSR-Seq). We showed that the *Pm* of Jimai 23 was provided by a single dominant gene, tentatively designated *PmJM23*, and assigned it to the documented *Pm2* region of chromosome 5DS. 3,816 consistently different SNPs were called between resistant and susceptible parents and the bulked pools derived from the combinations between the resistant parent Jimai23 and the susceptible parent Tainong18. 58 of the SNPs were assigned to the candidate region of *PmJM23*. Subsequently, 3,803 differentially expressed genes (DEGs) between parents and bulks were analyzed by GO, COG and KEGG pathway enrichment. The temporal expression patterns of associated genes following *Bgt* inoculation were further determined by RT-qPCR. Expression of six disease-related genes was investigated during *Bgt* infection and might serve as valuable genetic resources for the improvement of durable resistance to *Bgt*.

## Introduction

Bread wheat (*Triticum aestivum* L.) is one of the most important and widely planted crops worldwide. Producing a high and stable yield of wheat, however, is constantly challenged by a series of diseases. Wheat powdery mildew, caused by *Blumeria graminis* f. sp. *tritici* (*Bgt*), is a devastating disease, reaching epidemic levels in maritime or semi-continental climates (Morgounov et al., 2012; Mehta et al., 2018). Infection by powdery mildew will lead to a 10–15% yield reduction in general, but which sometimes can be as high as 62% in severely infected fields (Singh et al., 2016). Apart from yield reduction, decreases in quality are also recognized and have been commonly reported (Pál et al., 2013). In China, the epidemic area of wheat powdery mildew has been around six million hectares for the last 10 years.^1^ To control this disease and prevent epidemics, fungicides are often used, but drug resistance of *Bgt* is increasingly serious due to pathogenic variation (Manoharachary and Kunwar, 2014). Alongside drug resistance, environmental pollution and cost caused by the use of pesticides cannot be ignored (Saharan et al., 2019). Compared to the use of fungicides, the breeding and use of resistant cultivars is considered to be the most effective and environmentally friendly means of preventing disease epidemics (Selter et al., 2014).

To improve the resistance of wheat to powdery mildew, abundant resistance resources and identification of genes are essential. Up to now, 88 formally designated powdery mildew resistance (*Pm*) genes/alleles have been identified at 66 loci (*Pm1*-*Pm66*) (McIntosh et al., 2019; Li et al., 2020a). In addition, more than 30 temporarily designated *Pm* genes have also been reported and assigned to their corresponding wheat chromosomes (Li et al., 2020b).^2^ Most of these genes were derived from common wheat and its relatives (Ma et al., 2018, 2019; McIntosh et al., 2019).

There are two types of resistance to wheat powdery mildew: qualitative and quantitative. Qualitative resistance is common in many of the reported *Pm* genes. These genes follow Mendel’s law of segregation. In contrast, several of the resistance genes are quantitatively inherited, including *Pm38* (Spielmeyer et al., 2008), *Pm39* (Lillemo et al., 2008), *Pm46* (Herrera-Foessel et al., 2014), and *Pm54* (Hao et al., 2015). Qualitative resistance is often defeated after extended periods in production whereas quantitative resistance is rarely overcome. The underlying molecular mechanism of disease resistance needs to be clarified to support the rational use of the *Pm* genes. The mechanism for powdery mildew infection has been reported in grapevines (Fung et al., 2008), barley (Eckey et al., 2004), and *Arabidopsis thaliana* (Fauteux et al., 2006) using vitis GeneChip, cDNA-AFLP, and cDNA microarrays, respectively. But wheat powdery mildew is different from that of the plants mentioned above and relatively little is known regarding the molecular mechanism of powdery mildew resistance in wheat. Only individual genes, including *NAC* (NAM ATAF1/2 CUC2) and *MYB* (V-myb avian myeloblastosis viral oncogene homolog) transcription factors, have been analyzed and shown to play a role in the resistance process (Zhou et al., 2018; Zheng et al., 2019).

Jimai 23 is an elite wheat cultivar released in the Shandong province of China. It shows high level resistance to powdery mildew over its entire life cycle. In this study we confirmed that a *Pm2* allele confers the powdery mildew resistance in Jimai 23. Although a *Pm2*-related gene has been cloned, the *Pm2* locus has been shown to be a complex locus (Sánchez-Martín et al., 2016; Jin et al., 2018; Ma et al., 2018; Chen et al., 2019), and the resistance mechanism of this locus is even less clear during Bgt invasion. In order to dissect the composition of the *Pm2* locus and profile the expression of powdery mildew resistance genes in Jimai 23 when subjected to *Bgt* invasion we: (1) confirmed the candidate interval of the *Pm* gene by distribution of differential SNPs; (2) identified and classified differentially expressed genes (DEGs) at the whole-genome scale; and (3) selected several key genes mediating powdery mildew resistance in Jimai 23 and profiled their expression following *Bgt* inoculation. To achieve this, we used RNA sequencing (RNA-seq), bulked segregant RNA-seq (BSR-seq) and reverse transcriptase quantitative PCR (RT-qPCR). RNA-seq is an effective and low-cost method to comprehensively assess the gene expression profiles of the *Pm* genes after inoculation by *Bgt* isolates. This is because RNA-seq is not dependent on pre-existing databases of expressed genes and can provide an unbiased view of gene expression profiling (Pearce et al., 2015; Pankievicz et al., 2016). BSR-seq, which is a combination of RNA-seq and bulked segregant analysis (BSA), is an efficient method for both differential gene expression profiling and rapid gene/QTL mapping (Wang et al., 2017; Hao et al., 2019). Especially for the crop species with complex genomes, BSR-seq can also break through the adverse effects of the genome sequences and help to obtain relatively advantageous dissection (Liu et al., 2012; Trick et al., 2012; Wang et al., 2013, 2017, 2018; Ramirez-Gonzalez et al., 2015; Zhang et al., 2017).

## Materials and Methods

### Plant Materials

The wheat cultivar Jimai 23, bred by the Crop Research Institute, Shandong Academy of Agricultural Sciences, was used as the resistant parent against powdery mildew in this study. The susceptible wheat cultivar Tainong 18 was crossed with Jimai 23 to produce *F*_1_, *F*_2_, and *F*_2__:__3_ populations for genetic analysis and BSR-Seq analysis. Wheat cultivar Huixianhong, which we have previously shown to be susceptible to a range of *Bgt* isolates (Ma et al., 2018), was used as the susceptible control in phenotypic assessment experiments.

### Preparation of Samples for BSR-Seq

Jimai 23, Tainong 18, together with their derived *F*_1_, *F*_2_, and *F*_2__:__3_ progeny, were inoculated with *Bgt* isolate YT01 for phenotypic assessment. At the seedling stage, five seeds were sown in each cell using 128-cell rectangular trays in a growth chamber which was set at 20°C with a daily photoperiod of 14 h. For Jimai 23, Tainong 18 and their derived *F*_1_ hybrids, ten seeds of each one were sown in six cells. For the *F*_2_ population and *F*_2__:__3_ families, 300 seeds and 200 families (20 seeds for each *F*_2__:__3_ family) were sown for genetic analysis and preparation of the samples for BSR-Seq. The susceptible control, Huixianhong, was planted randomly in each tray. When the test seedlings had grown to the one leaf stage, they were inoculated with fresh conidiospores previously increased on Huixianhong seedlings, and immediately incubated in a dark and 100% humidity space at 18°C for 24 h, and then the growth chamber was set at 20°C with a daily photoperiod of 14 h. Over the next 2 days, the inoculation was conducted twice before dark. When the spores were fully developed on the first leaf of the susceptible control Huixianhong, which was at about 10–14 days after inoculation, infection types (ITs) were scored using the 0–4 scale described by An et al. (2013), in which ITs 0, 0, 1, and 2 are regarded as resistant, and ITs 3 and 4 as susceptible. Three parallel experiments were conducted using the same procedure to confirm the phenotypic data.

When the spores were fully developed on the first leaf of susceptible control Huixianhong, Total RNA of Jimai 23, Tainong 18, and 40 homozygous resistant and susceptible *F*_2__:__3_ families was isolated from the first leaf tissues using the Spectrum Plant Total RNA kit (Sigma-Aldrich) following the manufacturer’s protocol. Resistant and susceptible RNA bulks were constructed by separately mixing equal amounts of mRNA from the 40 homozygous resistant and susceptible *F*_2__;__3_ families, respectively.

### Library Construction and RNA Sequencing

The RNA integrity of Jimai 23, Tainong 18, and the resistant and susceptible bulks was evaluated using the Agilent 2100 Bio analyzer (Agilent Technologies, Santa Clara, CA, United States). Samples with an RNA Integrity Number (RIN) ≥ 7 were regarded as meeting the sequencing standard and cDNA libraries were constructed using TruSeq Stranded mRNA LTSample Prep Kit (Illumina, San Diego, CA, United States) according to the manufacturer’s instructions. The quality of the cDNA libraries was again assessed using the Agilent 2100 Bioanalyzer (Agilent Technologies, Santa Clara, CA, United States), and the acceptable cDNA libraries were sequenced on the Illumina HiSeq sequencing platform (Illumina HiSeq4000) by Beijing Biomics Technology Company Limited (Beijing, China). The sequencing indicator was set as 10 Gb clean data for parents and 20 Gb clean data for the two bulks. After the sequencing of the cDNA libraries, raw data was filtered, and joint sequences and poor-quality reads were eliminated to obtain high-quality clean data. The clean data were then assembled using the reference genome of Chinese Spring (The International Wheat Genome Sequencing Consortium, 2018) (v1.0) for subsequent SNP calling, differential gene expression, and GO and KEGG pathway analyses in Cloud Platform developed by Beijing Biomics Technology Company Limited.

### SNP Calling and BSR Association Mapping

The reads of Jimai 23, Tainong 18, resistant and susceptible bulks were aligned with the Chinese Spring reference genome (v1.0) using the STAR (v2.3.0e) software. SNP calling was done following the reference flowchart aimed at RNA-Seq by the software GATK (v3.1-1). SNP index values in the two bulks was calculated using MutMap method (Abe et al., 2012) with SNPs in the susceptible parent as a reference. Subsequently, a ΔSNP index between resistant and susceptible parents and bulks for each SNP was calculated (Takagi et al., 2013) using the following formula:

ΔSNP_index = (SNP_index of resistance parent/bulk) – (SNP_index of susceptible parent/bulk).

The average value of ΔSNP index in each window was calculated by sliding the window using a 5-Mb size as a step. The threshold for SNP screening was set as a test of 100,000 permutations in coupling with 99% confidence (Takagi et al., 2013). Candidate regions with higher confidence (99%) and SNPs with larger than the threshold ΔSNP index value (set as 0.75) in candidate regions were considered to be candidate loci related to powdery mildew resistance.

### DEGs Analysis

After clean reads were mapped to the reference genome, the expression level was calculated using FPKM (Fragments per kilo bases of exon per million fragments mapped) (Garber et al., 2011). DEGs were detected using Fold Change ≥ 2 and FDR (False discovery rate) < 0.01 as standard by the software EBSeq. Statistical significance of DEGs was determined using a combination of multiple tests and false discovery rate (FDR) (Reiner et al., 2003). Statistics and clustering analysis of DEGs between parents and bulks were done to present the expression pattern genome-wide, including the candidate interval.

### Functional Annotation and Enrichment Analysis of the DEGs

Functional annotation of the DEGs was performed using the IWGSC (International Wheat Genome Sequencing Consortium) database (v1.0). GO, COG, and KEGG pathway enrichment analyses were performed using an R package for DEGs (Yu et al., 2012). For GO analysis, GO Term Finder was used to describe the biological functions of a gene expression product (Boyle et al., 2004). For COG analysis, the Unigene sequences were aligned to the COG database to predict possible functions, and to determine the gene function distribution characteristics.^3^ For KEGG pathway analysis, the KEGG database was used to blast against the metabolic pathway.^4^

### Sample Preparation and RT-qPCR

RT-qPCR was performed to profile the expression of the DEGs in the targeted interval that may be related to the powdery mildew resistance in Jimai 23. In addition, several genes that were not differentially expressed, but which are potentially related to pathogen invasion, were selected for further investigation because the DEG analysis only targeted one time point after inoculation. Seedlings of Jimai 23 and Tainong 18 were inoculated with the *Bgt* isolate YT01 at the one-leaf stage. The first leaf of Jimai 23 and Tainong 18 seedlings was sampled 3, 6, 12, 24, 36, 48, and 72 h after inoculation. Three parallel experiments were set up at this stage. The leaves were immediately frozen in liquid nitrogen and ground to a fine powder in a pestle and mortar. RNA was extracted using the Spectrum Plant Total RNA kit (Sigma-Aldrich) following the manufacturer’s recommendations and quantified by measuring absorbance at the wavelengths of 260 and 280 nm using a Nano Drop 1000 spectrophotometer (Thermo Scientific). Finally, the RNA was DNase treated with Promega DNase I prior to cDNA synthesis.

One microgram of total RNA was used for cDNA synthesis using Invitrogen SuperScript-II reverse transcriptase following the manufacturer’s guidelines. RT-qPCR analysis was performed as described previously (He et al., 2016, 2018), using SYBR green master mix (Applied Biosystems) with a Rotor-Gene-Q (Qiagen). Amplification was followed by melt curve analysis. The 2^–ΔΔ*Ct*^ method was used for relative quantification (He et al., 2018). To detect transcript levels, primers for specific genes were designed based on the coding sequences of the selected genes (Supplementary Table S1). Oligonucleotides amplifying *ACTIN* were used for normalization.

## Results

### Powdery Mildew Resistance Evaluation and Genetic Analysis

When inoculated with *Bgt* isolate YT01, Jimai 23 showed no visible symptoms on the first leaf (IT 0), while Tainong 18 showed abundant sporulation with more than 80% of the leaf area covered with aerial hyphae. It was scored IT 4, susceptible. All the nine *F*_1_ plants of Jimai 23 × Tainong 18 were germinated and all showed resistance to YT01 with IT scores of 0. The 271 generated *F*_2_ plants segregated into 198 resistant and 73 susceptible ones, which is consistent with the theoretical ratio for monogenic segregation (χ^2^ = 0.54; *P* = 0.46). Two hundred *F*_2__:__3_ families further confirmed the ratio for monogenic segregation, with segregation ratio of 47 homozygous resistant (RR): 103 segregating (Rr): 50 homozygous susceptible (rr) families (χ^2^ = 0.27; *P* = 0.87) after YT01 infection. We therefore concluded that the seedling-resistance to *Bgt* isolate YT01 in Jimai 23 is controlled by a single dominant mendelian factor from the level of genetic analysis, tentatively designated *PmJM23*. Subsequently, mRNA from 40 randomly selected homozygous resistant and susceptible *F*_2__:__3_ families was isolated and pooled to make the resistant and susceptible bulks for subsequent RNA-Seq.

### Clean Data, Quality Control, and Sequence Alignments

After filtering low-quality reads and adaptors, there was more than 12 Gb clean data for each of the parents, Jimai 23 and Tainong 18, and more than 37 Gb clean data for each of the resistant and susceptible bulks. The data size greatly exceeded the transcript size of the wheat genome and it was, therefore, assumed to cover most transcribed genes within the wheat genome. The percentage of clean reads with a Q30 was greater than 94% for all four of the samples, and the GC content ranged from 48.92 to 56.44%. After aligning the four sets of clean reads to the reference genome (IWGSC v1.0) individually, the percentage of reads mapping to the reference genome ranged from 50.05 to 89.79% (Table 1). In summary, the sequencing quality of the four samples was high and suitable for subsequent analysis.

**TABLE 1:** Data summary of RNA-seq for Jimai 23, Tainong 18 and their derived resistant and susceptible bulks obtained from 40 homozygous resistant and 40 homozygous susceptible *F*_*2:3*_.

Samples	Total base pairs	Clean reads	GC content of clean reads (%)	Clean reads Q30 (%)	Genome map rate (%)
Jimai 23	13,244,236,200	44,147,454	56.44%	94.50%	89.79%
Tainong 18	12,632,022,300	42,106,741	50.89%	94.75%	56.42%
Resistance bulk	53,859,298,800	179,530,996	51.83%	95.67%	88.57%
Susceptible bulk	37,628,667,600	125,428,892	48.92%	94.58%	50.05%

### SNP Calling and Confirmation of Candidate Interval

**FIGURE 1:**
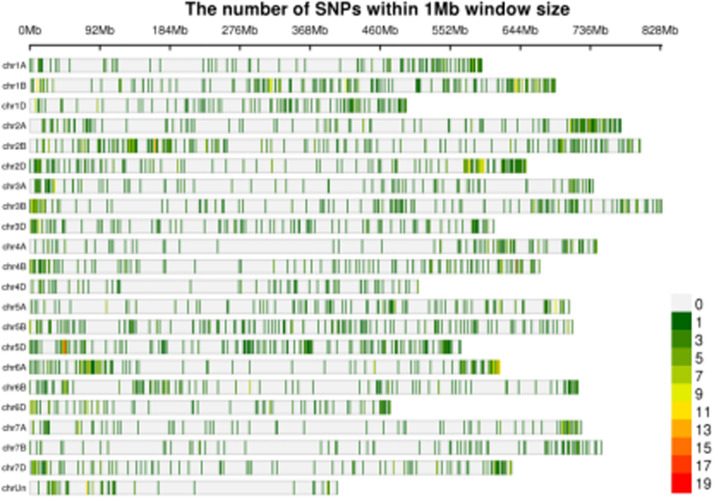
Distribution of the SNPs with consistent differences between the resistant parent Jimai23 and susceptible parent Tainong18 and their derived bulked pools on 21 chromosomes based on ΔSNP index value. Density indicating meter is shown as the color scale in the bottom right corner.

In order to confirm the candidate region within the wheat genome that was responding to powdery mildew infection, differential SNPs from the transcriptome data were screened. A total of 63,875 (Jimai 23), 237,919 (Tainong 18), 64,093 (Resistance bulk), and 77,438 (Susceptible bulk) SNPs were detected from the respective clean data. The three steps for SNP filtering were then carried out. Firstly, SNPs with support degree less than three were filtered; secondly, SNPs that were inconsistent between parent and corresponding bulk were filtered; thirdly, SNPs that were consistent between resistant and susceptible bulks were filtered. Finally, 3,816 SNPs with consistent differences between resistant and susceptible parents and bulks were obtained for subsequent ΔSNP index analysis (Figure 1 and Supplementary Table S2).

Using 99% confidence as the threshold, only one putative candidate region near the end of chromosome arm 5DS was identified (Figure 1). Multiple *Pm2* alleles have been reported in this region (Ma et al., 2018). To confirm this locus, *Cfd81*, the universal detection marker for different *Pm2* alleles, was used to genotype *F*_2__:__3_ families of Jimai 23 × Tainong 18. The result showed that *Cfd81* co-segregated with the *Pm* gene in Jimai 23, indicating that the *Pm* in Jimai 23 was also likely controlled by a *Pm2* allele. In this interval, more than 50 SNPs with consistent differences between resistance and susceptible parents and bulks revealed high confidence. These SNPs were used as the reference for the DEG analysis (Supplementary Table S2).

### Discovery and Classification of DEGs

**FIGURE 2:**
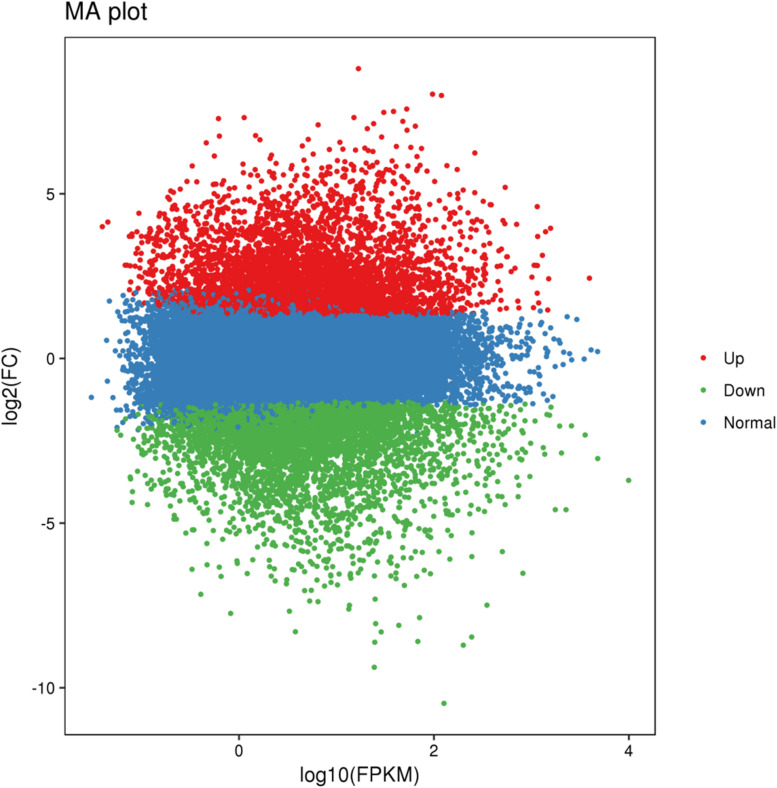
MA plot of the DEGs with consistent differences between the resistant parent Jimai23 and susceptible parent Tainong18 and their derived bulked pools. *X* and *Y* axes showed overall signal strength and output difference of the DEGs between resistant and susceptible parents and bulks.

Using BSR-Seq, a total of 124,200 genes was identified from the parents and bulks. Among them, 12,361 DEGs were detected between Jimai 23 and Tainong 18, of which, 5,822 DEGs were down-regulated and 6,539 DEGs were up-regulated, using the expression index of Tainong 18 as a standard. Furthermore, 9,162 DEGs were detected between the resistance and susceptible bulks, of which 4,606 and 4,496 DEGs were downregulated and up-regulated, respectively. For further screening, 3,803 DEGs showed consistent expression difference between parents and bulks (Figure 2 and Supplementary Table S3). Combined with the candidate interval analysis, only 16 DEGs were located in this interval (Supplementary Table S1). These genes were considered to be prime candidates in the resistance response of Jimai 23 to powdery mildew.

### GO, COG, and KEGG Pathway Significant Enrichment Analyses of DEGs

**FIGURE 3:**
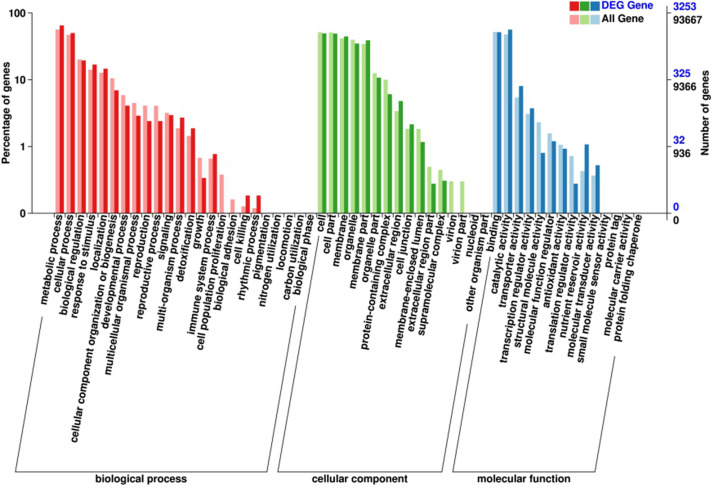
Gene ontology (GO) analysis of the DEGs with consistent differences between the resistant parent Jimai23 and susceptible parent Tainong18 and their derived bulked pools.

**FIGURE 4:**
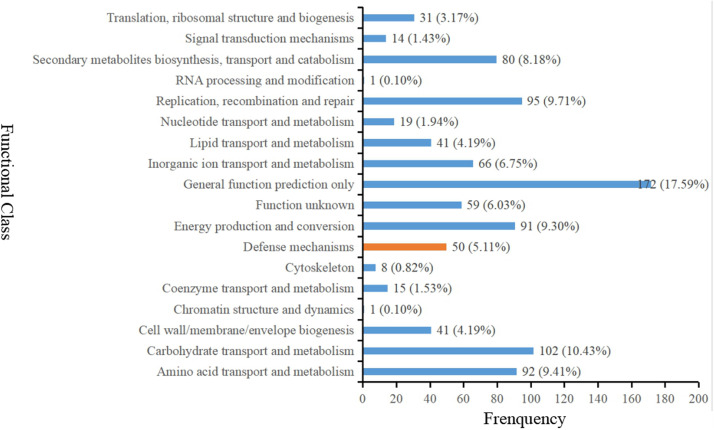
Clusters of orthologous groups (COG) analysis of the DEGs with consistent differences between the resistant parent Jimai23 and susceptible parent Tainong18 and their derived bulked pools.

Gene ontology (GO) analysis was firstly performed on the DEGs that showed consistent expression difference between parents and bulks via differential expression analysis. These DEGs were mainly involved in biological processes. These included: metabolic processes, cellular processes, biological regulation and response to stimulus; cell components including cell, cell part, membrane and organelle; and molecular functions comprising of binding catalytic and activity (Figure 3). However, the results of the GO analysis describe only the main processes exhibited after *Bgt* infection. Although the ‘response to stimuli’ process was significantly enriched and may directly participate in disease defense, no DEGs known to relate to defense mechanism(s) were detected. Therefore, clusters of orthologous groups (COG) analysis was performed using the same EDGs above. The data showed that the DEGs were mainly involved in transport and metabolic processes, such as amino acid and carbohydrate transport and metabolism, and DNA duplication, transcription, recombination and repair (Figure 4). However, a few DEGs were directly involved in plant defense, but accounting for only 1.44% of the DEGs. These results indicated that more genes related to biological metabolism and synthesis were activated to participate in biological defense rather than defense-related genes themselves. In other words, activation of defense mechanisms needs the support of biosynthesis and metabolism.

**FIGURE 5:**
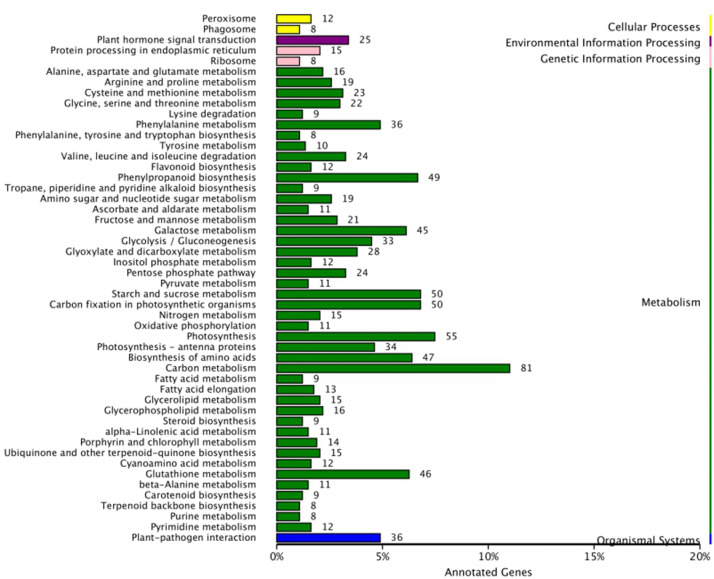
KEGG pathway analysis for DEGs with consistent differences between the resistant parent Jimai23 and susceptible parent Tainong18 and their derived bulked pools.

**FIGURE 6:**
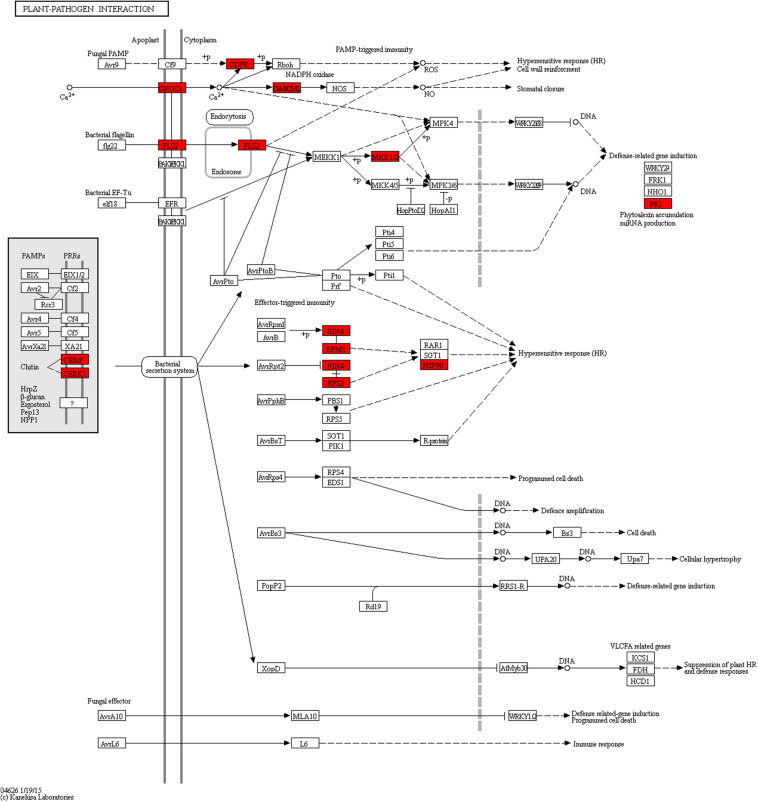
Plant–pathogen interaction pathway enriched from DEGs with consistent differences between the resistant parent Jimai23 and susceptible parent Tainong18 and their derived bulked pools. Red indications show the DEGs from this study.

To further investigate the signal transduction pathway(s) that the DEGs may be involved in, significance enrichment analysis for KEGG pathway was performed on the DEGs that showed consistent expression differences between parents and bulks in the differential expression analysis. Hundred and three significantly enriched (*Q* ≤ 0.05) pathways involving 50 categories in cellular processes, environmental processing, genetic information processing, metabolism and organismal system were found (Figure 5). Among them, one plant-pathogen interaction pathway was enriched, and 14 DEGs were present in this pathway. These genes are a resource for further molecular studies into the plant response to powdery mildew (Figure 6).

### RT-qPCR Verification for the Disease-Resistance Related Genes in Jimai 23

To profile the expression of the disease resistance-related genes in Jimai 23, we monitored the transcriptional level of 21 potential target genes (including 16 DEGs in the candidate interval) (Supplementary Table S1) at different stages after inoculation with *Bgt* isolate YT01. Six of the target genes showed significant differences between the resistant Jimai 23 and the susceptible Tainong 18 in the time course analysis following *Bgt* inoculation.

**FIGURE 7:**
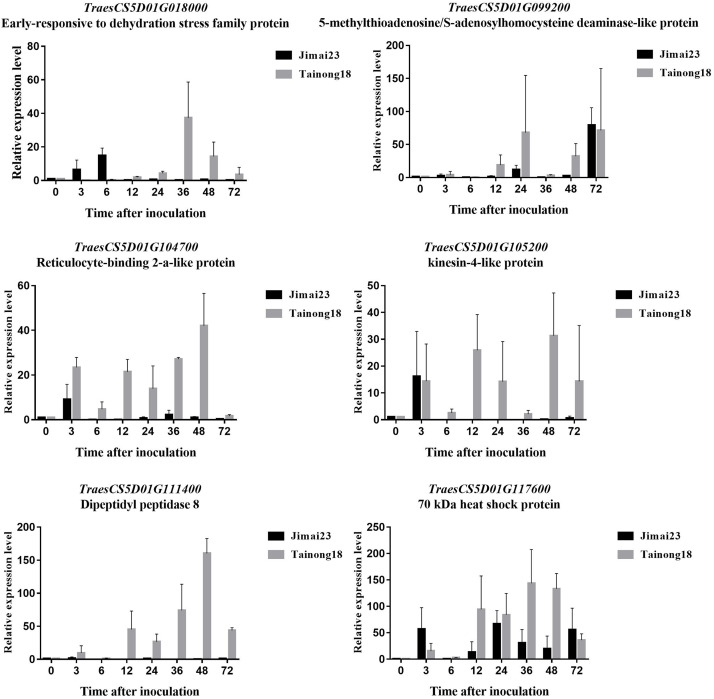
Expression profiles of TraesCS5D01G018000 **(A)**, *TraesCS5D01G117600* **(B)**, *TraesCS5D01G104700* **(C)**, *TraesCS5D01G105200* **(D)**, *TraesCS5D01G099200* **(E)**, and *TraesCS5D01G111400* **(F)** in each corresponding stage of Jimai 23 and Tainong 18 after *Bgt* infection.

The transcriptional levels of two genes [*TraesCS5D01G018000* encoding an early-responsive to dehydration (ERD) stress family protein and *TraesCS5D01G117600* encoding a 70 kDa heat shock protein (HSP70)] were rapidly up-regulated in Jimai 23 but not in Tainong 18 within 0–6 h after inoculation (Figures 7A,B). Their expression was highly induced only in Tainong 18 after 36 h (Figures 7A,B). *TraesCS5D01G104700* and *TraesCS5D01G105200* encode a reticulocyte-binding 2-a-like protein and a kinesin-related protein, respectively. The transcriptional levels of these two genes were initially both up-regulated in Jimai 23 and Tainong 18, but then elevated expression was maintained only in Tainong 18 (Figures 7C,D). *TraesCS5D01G099200* (encoding a *S*-adenosyl homocysteine deaminase-like protein) and *TraesCS5D01G111400* (encoding a dipeptidyl peptidase protein) were only up-regulated in Tainong 18 during infection.

## Discussion

Jimai 23 is an elite wheat cultivar released in the Shandong Province of China that is highly resistant to powdery mildew. In this study, through BSR-Seq combined genetic analysis, a dominant allele of *Pm2* was confirmed to control the resistance in Jimai 23. In our previous reports, a series of *Pm2* alleles with different reaction patterns to *Bgt* isolates have been identified in various wheat genotypes (Ma et al., 2014, 2015a,b,c, 2016, 2018; Xu et al., 2015; Jin et al., 2018). However, these *Pm2* alleles were only characterized at the genetic level. Even though a *Pm2-*related gene was cloned using mutant chromosome sequencing and candidate gene analysis using the reference genome of Chinese Spring (Sánchez-Martín et al., 2016; Chen et al., 2019), there are still aspects of this locus that require further investigation. For instance, while the homologous sequences of different *Pm2* alleles are identical to each other, these alleles have significantly different reaction patterns to *Bgt* isolates with different virulence spectra, something which cannot be explained by the background difference of the wheat genotypes (Jin et al., 2018; Ma et al., 2018).

Meanwhile, we determined the expression levels of 21 potential target genes in the *Pm2* candidate interval, but only six genes were differently expressed between Jimai 23 and Tainong 18. This further suggest that the *Pm2* locus in Jimai 23 is most likely a more complex and larger interval compared to that shown within the reference genome of common wheat Chinese Spring, and that the powdery mildew resistance is most likely conferred by multiple genes from the level of gene analysis. So, in-depth study on the composition of the *Pm2* locus and dissection of its molecular mechanism is imperative. Surrounding the problems above, an expression profiling dissection was conducted on Jimai 23 using BSR-Seq in the present study, which is a high efficiency and low-cost means to investigate the overall expression profile of resistance-related genes (Hao et al., 2019). A large number of DEGs, including the target genes in the candidate interval which are important for defense against *Bgt* invasion, were identified and analyzed using GO, COG, and KEGG enrichment.

Plant resistance is a complex process in the course of host-pathogen interaction (Hikichi, 2016). From the host’s perspective, a mass of genes will be activated in response to the intrusion of external pathogens. Entry of the pathogen could be prevented at different layers, such as cell wall, plasma membrane and various enzymes in cytoplasm (Braeken et al., 2008; Soto et al., 2011; Siddiqui et al., 2012). However, there are relatively few studies of the regulatory mechanism within wheat in response to powdery mildew, and, so far, only the functional mechanism of individual genes has been analyzed during powdery mildew infection, including *MYB* and *NAC* transcription factors (Zhou et al., 2018; Zheng et al., 2019). From the BSR-Seq analysis, we selected 21 target genes for consideration at different stages of pathogen invasion. These genes included structural protein, translocator, regulatory protein and stress response protein (Supplementary Table S1), indicating an overall response model from structure change, biosynthesis and transport, and direct stress response after *Bgt* invasion. It should also be pointed out that the sampling for BSR-Seq was only from the stage at which disease symptoms were visible, and did not represent the early stages of infection, so these genes were selected not solely based on differential expression but also on the functional analysis of the genes in the candidate interval. We then profiled the expression of the six of the 21 selected genes following pathogen inoculation to analyze their response to powdery mildew in resistant versus susceptible cultivars.

*TraesCS5D01G018000* encoded an ERD protein that is related to plant adaptation to stress conditions. The ERD families have been reported to provide enhanced drought and salt tolerance and respond to abscisic acid treatment in *Arabidopsis*, sugarcane and maize (Liu et al., 2009; Rai et al., 2016; Devi et al., 2019), suggesting the key role of the ERD protein families is in plant stress tolerance or resistance. The *TraesCS5D01G117600*-encoded HSP70 is one of the members of the HSP70 family, which are well known as stress responsive molecular chaperones involved in the correct folding of newly synthesized proteins. Although they were first identified in the heat stress response, the heat shock proteins have also been reported to play key roles in innate immunity responses, and to be essential for the functioning of other resistance proteins (Neckers and Tatu, 2008). For wheat, preliminary proteomic analysis also revealed that HSP70 may be involved in regulation of resistance to powdery mildew (Mandal et al., 2014), which aligns with our findings. These two genes were rapidly up-regulated in Jimai 23 at the early stage following *Bgt* inoculation, whereas in Tainong 18, they were induced only after 36h. We suggest that early defense activation of the two genes in Jimai 23 may be key to its resistance to powdery mildew. In Tainong 18, *Bgt* may have broken through any early defense barriers and the elevated expression at the later stage in Tainong 18 was no longer effective, leading to the sensitivity of Tainong 18 to powdery mildew.

In addition to the key defensive proteins mentioned above, we also selected two genes (*TraesCS5D01G104700* and *TraesCS5D01G105200*) encoding a structural protein and kinesin, respectively, for investigating cell structure changes after *Bgt* infection. The expression of these two genes were both up-regulated in Jimai 23 and Tainong 18 rapidly after *Bgt* inoculation, but remained elevated only in susceptible Tainong 18, suggesting that the attack of *Bgt* was blocked in Jimai 23 whereas it was aggravated in Tainong 18 within 6 h after inoculation, which is consistent with the expression of *TraesCS5D01G018000* and *TraesCS5D01G117600*. One reasonable explanation is that these transcriptional changes may lead structural change of the cell and be a consequence of the resistance, but not its cause. Changes in other structural proteins was also reported in wheat line N0308 after infection by *Bgt* using proteomic analysis (Mandal et al., 2014).

*TraesCS5D01G099200* encoded an *S*-adenosyl homocysteine deaminase-like protein which is an intracellular oxidation-reduction enzyme (Meisel et al., 1980). *TraesCS5D01G111400* is a dipeptidyl peptidase (Park et al., 2006). These two genes have been reported to be involved in the plant resistance pathway within the cell, with the *S*-adenosyl homocysteine deaminase-like protein mediating disease resistance through the methionine cycle (Meisel et al., 1980; Park et al., 2006; Mäkinen and De, 2019), and dipeptidyl peptidase serving as a bio-marker of disease (Yazbeck et al., 2018). The transcription of these two genes was only up-regulated in Tainong 18 after *Bgt* infection, suggesting the defense system of susceptible Tainong 18 was vulnerable. We suggest that the activation of these two genes was not observed following *Bgt* inoculation of Jimai 23, as the pathogen did not invade its cells.

## Conclusion

We investigated the holistic expression profile responding to powdery mildew in the resistant wheat cultivar Jimai 23, and six key genes potentially involved in the resistance process were further investigated following *Bgt* inoculation. Compared with previous reports, where the focus was mainly on the mechanism of a single gene, our study provided a perspective of overall expression profiling, which can facilitate dissection of resistance pathways and accelerate improvement of durable resistance. The selection of potential key genes in the present study mainly focused on the candidate interval of *PmJM23*. In the future, we will select more genes in other intervals, especially from the plant pathogen interaction pathway, for a deeper dissection of the resistance mechanism.

## Data Availability Statement

We have uploaded the sequencing data to NCBI and the BioProject ID is PRJNA625022.

## Author Contributions

PM, CL, and HX conceived the research. TZ, LW, and MJ performed the experiments. HH, JS, LL, HL, JL, LN, WD, XW, and RH analyzed the data. XZ, WW, and XL performed the phenotypic assessment. TZ and PM wrote the manuscript. All authors read and approved the final manuscript.

## Conflict of Interest

The authors declare that the research was conducted in the absence of any commercial or financial relationships that could be construed as a potential conflict of interest.

## Footnotes

1 https://www.natesc.org.cn/

2 https://shigen.nig.ac.jp/wheat/komugi/genes/symbolClassList.jsp

3 http://www.ncbi.nlm.nih.gov/COG

4 https://www.kegg.jp/kegg/pathway.html
